# Microstructure, Digestibility and Physicochemical Properties of Rice Grains after Radio Frequency Treatment

**DOI:** 10.3390/foods11121723

**Published:** 2022-06-13

**Authors:** Zhenna Zhang, Bin Zhang, Lin Zhu, Wei Zhao

**Affiliations:** 1 School of Food Science and Technology, Jiangnan University, Wuxi 214122, China; zhangzhenna321@163.com (Z.Z.); 15617571473@163.com (B.Z.); 2 Key Laboratory of Preservation Engineering of Agricultural Products, Institute of Agricultural Products Processing, Ningbo Academy of Agricultural Sciences, Ningbo 315040, China; zhulin0822@163.com

**Keywords:** rice, radio frequency, microstructure, physicochemical properties, digestibility

## Abstract

Radio frequency (RF) energy has been successfully applied to rice drying, sterilization, and controlling pests. However, the effects of RF treatment on the microstructure, physicochemical properties, and digestibility of rice have rarely been studied. This study investigated the alteration of a multiscale structure, pasting, rheology, and digestibility of rice grains after the RF treatment. A microstructure analysis demonstrated that the RF treatment caused starch gelatinization and protein denaturation in rice grains with an increasing treatment time. After the RF treatment, indica and japonica rice (IR and JR) remained as A-type crystals, with the formation of an amylose–lipid complex. In contrast, the crystalline structure of waxy rice (WR) was disrupted. The RF treatment led to a decrease in crystallinity and short-range ordered structures. However, the DSC results indicated that the RF treatment enhanced the T_o_, T_p_, and T_c_ of IR and JR. The RF treatment resulted in an increase in the resistant starch (RS) of IR and JR, thereby reducing the digestibility. In addition, the pasting profiles of IR and JR after RF treatment were reduced with the increase in treatment time, while the RF-treated WR showed an opposite trend. The storage modulus (G′) and loss modulus (G″) of all samples after the RF treatment obviously increased compared to the control.

## 1. Introduction

Rice (*Oryza sativa* L.) is widely cultivated and consumed by humans worldwide, as it plays a vital role in human nutrition, energy supply, and food security [1]. Compared with other cereals, rice is already classified as a high-glycemic-index (GI) food, owing to its higher digestible energy [2]. The high-GI food is correlated with diet-associated diseases such as type II diabetes, obesity, and cardiovascular disorders [3], which pose a threat to human health. Therefore, there is a need to develop low-GI rice to improve human health.

Various methods have been applied to modify rice GI by manipulating the starch structure to reduce the rate of starch digestion. Depending on the rate of digestion, starch is generally divided into rapidly digestible starch (RDS), slowly digestible starch (SDS), and resistant starch (RS) [4]. SDS and RS have been considered beneficial for the control of postprandial blood glucose, thereby preventing the occurrence of these metabolic diseases [5,6], and eventually displaying positive health benefits. Hence, increasing the SDS or RS contents of rice to satisfy the requirements of consumers has attracted more attention. Compared to the chemical and enzymatic modification of starch, physical methods are preferred by consumers, as it is natural and highly safe [7].

Radio frequency (RF) heating has been successfully used in food processing, such as drying, thawing, disinfestations, pasteurization, blanching, and roasting [8]. It is regarded as a promising alternative to replace conventional thermal methods due to faster heating and a higher penetration depth. During RF heating, electromagnetic energy is converted into thermal energy by dipole rotation and ionic migration in food [8]. The application of RF heating in rice mainly focused on insect control and storage [9,10,11]. However, as far as we know, the effects of RF treatment on the microstructure, physicochemical properties, and digestibility of rice grain have rarely been investigated. Our previous study showed that the RS content of rice flour was enhanced after RF treatment, leading to the strengthening of its resistance to enzyme hydrolysis [12]. White rice is composed of starch, protein, lipids, and non-starch polysaccharides. The starch properties and starch–protein/lipid interactions in rice grains play a critical role in improving digestibility. For example, Gong et al. [13] reported that rice starches with high short- and medium-amylose chains accelerated short-term retrogradation, thereby reducing the digestibility of starch. Li et al. [14] showed that the perfect arrangement of amylopectin double helices was conducive for the starch to resist the digestion of amylase. Moreover, it has been demonstrated that the interaction between starch and protein/lipids in rice remarkably affects starch digestibility [15,16]. Therefore, the microstructure of rice grains during RF treatment should be investigated to provide sufficient knowledge for industry application.

Hence, this work investigates the effect of the RF treatment on the microstructure, physicochemical, and digestive characteristics of different rice varieties. The results suggest that RF technology can be applied to reduce the digestibility of rice as an alternative to conventional methods and provide a theoretical basis for industry applications.

## 2. Materials and Methods

### 2.1. Materials

Three rice samples, including indica (Simiao), japonica (Daohuaxiang 2), and waxy (lvnuo 619) rice (IR, JR, and WR), used in this study were obtained from a supermarket in Wuxi (Jiangsu, China). Pepsin (P7000), pancreatin (P7545), and amyloglucosidase (A7095) were provided by Sigma-Aldrich (Shanghai, China). The Glucose oxidase-peroxidase kit was obtained from the Nanjing Jiancheng Bioengineering Institute (Nanjing, China). The other chemical reagents used in this work were of analytical grade.

### 2.2. Sample Preparation

Rice grains were soaked in distilled water (1:3 *w*/*v*) and equilibrated at room temperature for 6 h. After soaking, the water was decanted, and the sample was drained. The moisture content of the sample was approximately 30%. Subsequently, these samples were subjected to RF system.

### 2.3. RF Treatment

**Figure 1:**
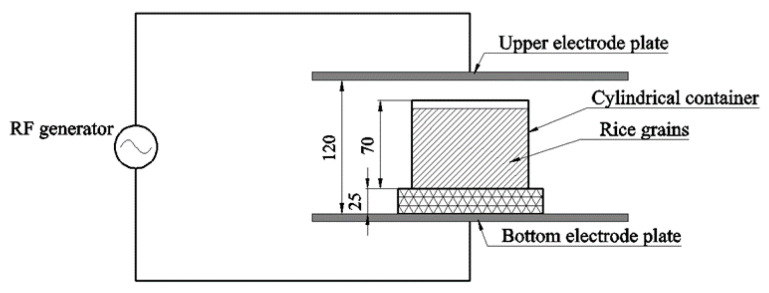
Schematic of the sample placed in the RF heating system (27.12 MHz, 6 kW). All dimensions are in mm.

An RF system with 6 kW, 27.12 MHz (GJG-2.1-10A-JY; Hebei Huashijiyuan High Frequency Equipment Co., Ltd., Shijiazhuang, China) was applied in this study for the modification of rice, as shown in Figure 1. According to our previous studies, the electrode gap was fixed at 120 mm for the whole experiment. The preprepared rice (250 g) was put into a cylindrical container, which was treated by the RF system at 120 mm for various times (10, 20, and 30 min). The temperature of samples was detected and collected by an optical fiber sensor. The RF-treated samples were cooled to 25 °C and then equilibrated at 4 °C for 48 h. After that, samples were dried, ground, and passed through a 100-mesh sieve for further analysis. The rice with different RF treatment times was tagged as IR-RF-10, IR-RF-20, IR-RF-30, JR-RF-10, JR-RF-20, JR-RF-30, WR-RF-10, WR-RF-20, and WR-RF-30.

### 2.4. Scanning Electron Microscopy (SEM)

SEM (SU8100, Hitachi, Ltd., Tokyo, Japan) was performed to analyze the microstructure of rice grains before and after RF treatment. According to the method of Zhong et al. [17], a razor blade was used to apply pressure to the cross-sectional axis of the rice grain to naturally fracture the rice grain. Then, the fractured rice grain was mounted on a stub using double-sided carbon-coated tape and was coated with gold. The micrographs of the fractured surface were collected at an accelerating voltage of 3.0 kV with different magnifications.

### 2.5. X-ray Diffraction (XRD)

The XRD patterns of samples were measured with an X-ray diffractometer (D2 PHASER, Bruker AXS Inc., Karlsruhe, Germany). The experimental conditions were as follows: scanning range of 5–40°, step size of 0.05°, and step duration of 0.5 s. Jade 6 software was used for analyzing the crystallinity of samples.

### 2.6. Fourier-Transform Infrared Spectroscopy (FTIR)

An FTIR spectrometer (IS10, Nicolet, WI, USA) was applied to detect the chemical structures of samples. Generally, samples were fully ground with KBr (1:100, *w*/*w*) and then compressed into tablets. The prepared sample was scanned from 4000 to 400 cm^−1^ with a resolution of 4 cm^−1^.

### 2.7. Differential Scanning Calorimetry (DSC)

The thermal properties of native and RF-treated rice were determined with DSC3 (Mettler-Toledo, Switzerland) following the method of Zhang et al. [18]. Approximately 3 mg samples and 6 μL deionized water were placed in a 40 μL aluminum pan, which was tested from 25 to 100 °C at a heating rate of 10 °C/min. The relative parameters, including onset temperature (T_o_), peak temperature (T_p_), conclusion temperature (T_c_), and gelatinization enthalpy (ΔH), were obtained.

### 2.8. Pasting Properties

Rapid viscosity analyzer (Newport Scientific Pty. Ltd., Sidney, Australia) was used to characterize the pasting properties of samples. First, 3 g samples and 25 g deionized water were placed in an aluminum canister. After that, the prepared sample was measured using the STD 2 procedure based on the method of Ma et al. [19]. The relative pasting parameters, including peak viscosity (PV), trough viscosity (TV), breakdown (BD), final viscosity (FV), and setback (SB), were collected.

### 2.9. Rheological Properties

Discovery Hybrid Rheometer-3 (TA instruments, New Castle, DE, USA) was applied to evaluate the rheological properties of samples, following the method of Zhang et al. [18]. After pasting properties measurement, the gelatinized samples were cooled to 25 °C and then placed on the testing platform equipped with a 40 mm parallel plate at a gap of 1 mm for rheological measurements. The dynamic frequency sweeps were tested from 0.1 to 100 rad/s at 0.5% strain. Storage modulus (G′), loss modulus (G″), and loss tangent (tan δ = G″/G′) of samples were determined.

### 2.10. In Vitro Digestibility

The in vitro digestibility of native and RF-treated rice grains was carried out as described by Englyst et al. [4] with certain modifications. Firstly, 200 mg samples were fully mixed with 10 mL sodium acetate buffer (pH 5.2, 0.1 mol/L) at room temperature. Then, they were heated for 30 min in a water bath at 95 °C with magnetic stirring. After that, the mixture was cooled down to 37 °C and equilibrated for 10 min. Afterwards, it was mixed with pepsin solution (10 mL, 5 mg/mL) and incubated for 30 min in a shaking water bath (180 rpm) at 37 °C. Next, 5 mL of sodium acetate buffer was added to the reaction mixtures and incubated for another 30 min. Thereafter, 5 mL mixed enzyme solution containing pancreatin and amyloglucosidase was added to each sample and allowed to react in a shaking water bath at 37 °C for 120 min. At various time points (0, 20, and 120 min), the hydrolyzed solution (0.1 mL) was collected and put into a centrifuge tube containing anhydrous ethanol (0.9 mL). After centrifugation at 5000 r/min for 10 min, the hydrolyzed glucose content in the supernatant was analyzed with a glucose oxidase-peroxidase kit. The RDS, SDS, and RS contents were calculated using the following equation:

RDS (%) = (G_20_ − G_0_) × 0.9 × 100/TS (1)

SDS (%) = (G_120_ − G_20_) × 0.9 × 100/TS (2)

RS (%) = (1 − RDS − SDS) × 100(3)

where G_0_, G_20_, and G_120_ reflect the mass of glucose released after enzymatic hydrolysis for 0, 20, and 120 min, respectively, and TS is the total starch content.

### 2.11. Statistical Analysis

All tests were performed at least in triplicate. Statistical analysis was conducted with Minitab 18 software (State College, PA, USA). Data were subjected to one-way analysis of variance (ANOVA) and the Tukey test at the 95% confidence level. Results were exhibited as means with standard deviations.

## 3. Results and Discussion

### 3.1. Microstructure

**Figure 2:**
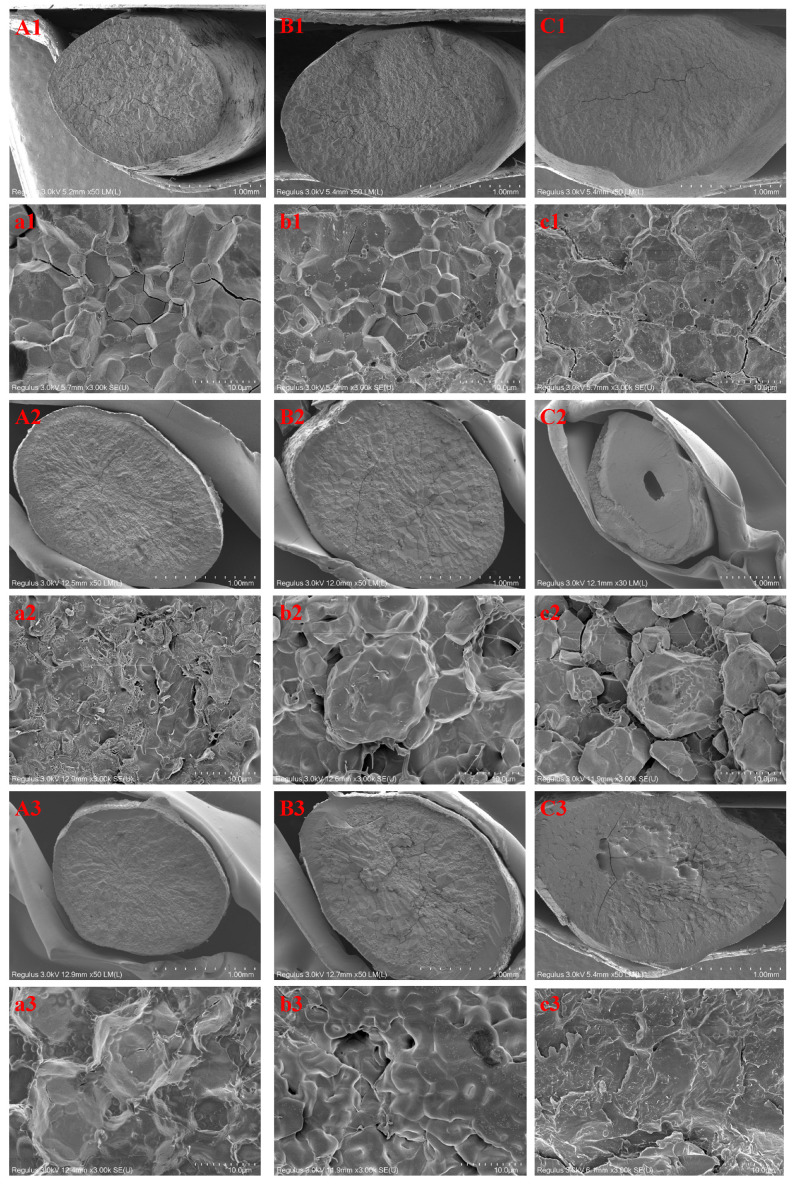

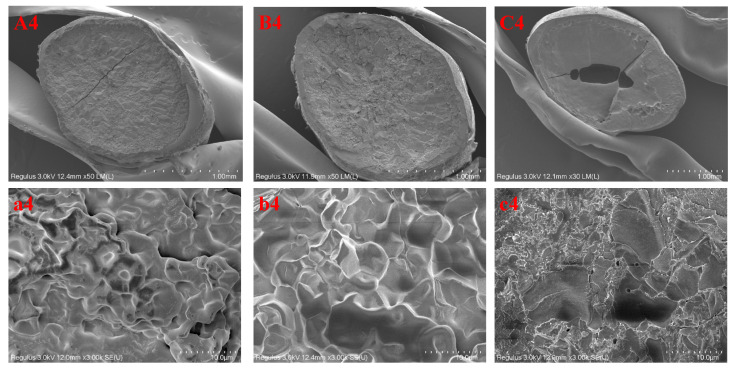
SEM images of different varieties of rice grains (indica, japonica, and waxy rice, coded “(**A**)/(**a**), (**B**)/(**b**), and (**C**)/(**c**)”, respectively) subjected to RF treatment at different treatment times (0, 10, 20, and 30 min, coded “1, 2, 3, and 4”, respectively). (**A**–**C**): the general profile of the cross-section; (**a**–**c**): partially enlarged view.

Figure 2 exhibits the transverse-section SEM images of native and RF-treated rice grains. They showed that the cross-section of the rice grains exhibited some cracks due to intercellular cleavage, and intracellular cleavage simultaneously occurred when the grains were cracked open [17]. In native rice, the starch granules were polygonal and were closely packed (Figure 2(a1–c1)). The surface layer of native rice was rough, with small protein molecules scattered on the surface and embedded in gaps between starch granules [20]. After RF treatment, the starch granules lost their angular character with the increasing RF treatment time, especially for RF-30. This could be attributed to the RF treatment inducing a high temperature in rice grains, resulting in partial starch gelatinization (Figure 2(a3–c3)). It has been reported that changes in rice grain structure happen mainly due to the gelatinization of starch [21]. Moreover, the protein structure in rice could be disrupted by the RF treatment, which could reconstruct and interact with starch, thereby altering the internal structure of the rice [22]. Furthermore, the starch granules in rice grains after the RF treatment swelled compared with those in native rice (Figure 2(a2–c2)). This phenomenon may have resulted due to the increased interior temperature, resulting in an increased interior pressure, which led to starch swelling and even gelatinization [23,24]. In addition, a hole appeared in the center of the WR subjected to the RF treatment, implying that the core of the RF-treated WR was overheated, causing either endosperm shrinkage or thermal disintegration [24].

### 3.2. Crystalline Structure

**Figure 3:**
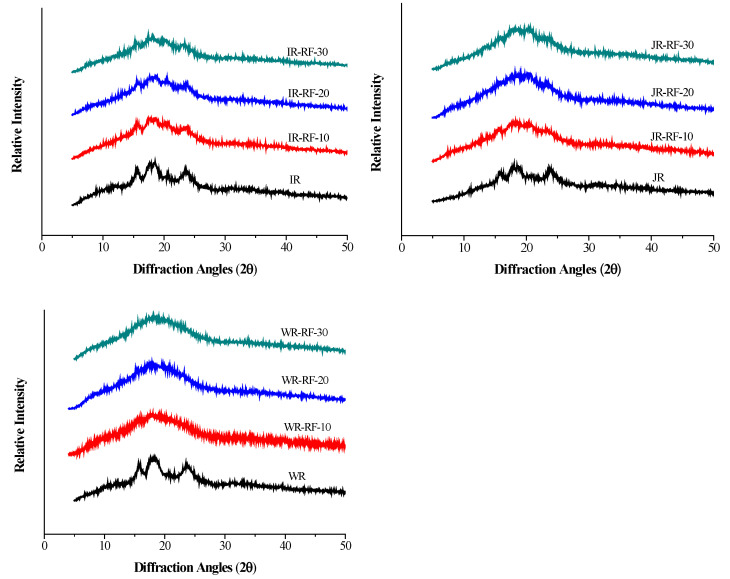
X-ray diffractograms of rice grains were submitted to RF treatment at different times (10, 20, and 30 min). IR: indica rice; JR: japonica rice; WR: waxy rice.

The X-ray diffractograms of untreated and RF-treated IR, JR, and WR are exhibited in Figure 3. The corresponding relative crystallinity is summarized in Table 1, which is generally applied to assess the long-range crystalline structure of rice [17]. All the native rice exhibited a typical A-type crystalline pattern, with main peaks at 2θ of 15°, 17°, 18°, and 23° [18]. After the RF treatment, the XRD patterns of IR and JR remained with a weakened diffraction intensity compared to the control. Moreover, there was a peak at 20° of the RF-treated IR and JR, suggesting the formation of amylose–lipid complexes (V-type pattern). With the increase in RF treatment time, the decrease in diffraction intensity was consistent with the presence of gelatinized starch [18]. Especially for WR, the typical diffraction peaks almost disappeared with a single peak after the RF treatment, implying that starch granules were in gelatinized and fused states.

On the other hand, the relative crystallinity of RF-treated rice grains was reduced compared to the control. It further confirmed that the RF treatment resulted in starch gelatinization and crystallite destruction. The reduction in RC may be ascribed to the friction and collision that occurred among polar molecules, rapidly producing more heat in rice grains, causing the destruction of starch granules and the degradation of molecular structures. In addition, the increased mobility of starch chains during the RF treatment also induced the disruption of crystalline regions [7]. Different changes of RC among the three rice grains indicated that the amylose content played a critical role in crystallite formation [20].

### 3.3. Short-Range Ordered Structure

The short-range ordered structure (single- and double-helical structures) of all samples was analyzed by FTIR in the range of 4000 to 500 cm^−1^. Generally, the absorption bands at 1022 and 1047cm^−1^ are related to the amorphous and crystalline regions of starch, respectively, and the alteration of ordered structures can be evaluated by the values of 1047/1022 cm^−1^ [25]. Moreover, the absorption band at 995 cm^−1^ is associated with C-OH bending vibrations [26], and the 1022/995 cm^−1^ is used to reveal the degree of double helices [27]. As shown in Table 1, the RF-treated samples exhibited lower values of 1047/1022 cm^−1^ than that of the control, except for WR-RF-10 and WR-RF-20. It signified that the RF treatment reduced the degree of ordered structures of starch at the short-range level, which might be ascribed to the partial gelatinization of starch after the RF treatment. The high temperature produced by the RF system might have disrupted the amorphous and crystalline structures of starch, resulting in the destruction of the packing or winding of the helical structures [28]. In addition, the changes of 1022/995 cm^−1^ values were opposite to the 1047/1022 cm^−1^ values. The RF-treated samples showed higher values of 1022/995 cm^−1^ than the untreated samples. The increase in 1022/995 cm^−1^ values after the RF treatment suggested the destruction of single- and double-helical structures, which were in agreement with XRD results.

### 3.4. Thermal Properties

The thermal properties of native and RF-treated rice grains are summarized in Table 1. The thermal properties of all the samples were significantly affected by the RF treatment, especially the RF treatment time. After the RF treatment, the T_o_, T_p_, and T_c_ of IR and JR were transferred to higher temperatures compared to the untreated sample. The increased T_o_, T_p_, and T_c_ of the RF-treated samples indicated that the RF treatment reinforced the interactions between the starch chains or starch and other components. Similar results were observed by Zhong et al. [29], showing that microwave irradiation enhanced the T_o_ and T_p_ of rice. Moreover, the higher gelatinization temperature of RF-treated samples implied that more energy was needed when the initial gelatinization of samples occurred. Changes in gelatinization temperature were mainly attributed to the amylose content and the amylopectin fine structure [30]. The higher gelatinization temperature of RF-treated IR could be primarily due to the higher amylose content. Furthermore, the gelatinization temperature of IR and JR increased with the increasing RF treatment time, suggesting that the RF treatment could improve the interaction among starch chains. However, compared with the untreated samples, the ΔH significantly decreased for the RF-treated samples, implying that the content of double-helical structures was reduced. This could be due to the partial gelatinization of rice grains caused by the RF treatment, but to a different extent [31]. In addition, the T_o_, T_p_, T_c_, and ΔH of the RF-treated WR decreased compared with the native sample. As revealed by the XRD and SEM results, the RF treatment seriously destroyed the microstructure of the WR.

### 3.5. Pasting Properties

**Figure 4:**
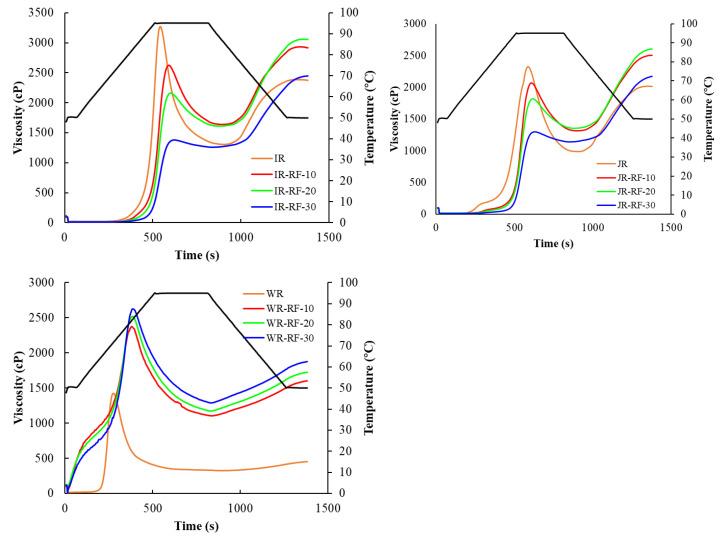
Pasting profile of native and RF-treated rice at different treatment times (10, 20, and 30 min). The black line corresponds to temperature. IR: indica rice; JR: japonica rice; WR: waxy rice.

Pasting properties are one of the most sensitive indicators in rice grains, responsible for the rice cooking and eating quality. The typical pasting curves of all samples are displayed in Figure 4, and the corresponding related parameters are presented in Table 2. Obviously, the pasting properties of the RF-treated rice grains were different from those of the untreated rice. This may be attributed to the degradation of starch and protein, the alteration of microstructure and intermolecular forces, and the enhanced hydrolytic enzyme activities of the RF-treated rice [22].

The pasting profiles of IR and JR after the RF treatment were similar to that of the untreated samples. As shown in Figure 4, the gelatinization time of the RF-treated IR and JR was delayed, meaning that the RF treatment had inhibiting effects on starch gelatinization. Moreover, the RF treatment caused a decrease in the PV and BD of IR and JR, whereas it enhanced the TV, FV, and SB. The PV of IR and JR significantly decreased with the increase in RF treatment time. The reduction in PV in IR and JR might have been due to the disruption of hydrogen bonds and glycosidic linkages [32] caused by the high temperature during the RF treatment. A similar phenomenon for the PV of rice after a microwave treatment was reported [33]. The high temperature affected the starch lamellar structure, resulting in gelatinization and a decreased paste viscosity. The decreased BD of the RF-treated IR and JR indicated that the RF treatment improved the thermal stability of starches with respect to the control sample. This result was in agreement with Sun et al. [34], suggesting that a heat moisture treatment reduced the BD of early indica rice. However, Zhong et al. [17] found that the microwave treatment enhanced the PV and BD of rice. The different changes in pasting properties may be due to the experiment conditions and rice varieties. Moreover, the higher SB of the RF-treated IR and JR implied that the recrystallization of amylose molecules increased the chance of starch retrogradation [35]. RF energy generated high internal thermal pressures in rice grains, which probably destroyed the cell wall and disintegrated the starch granules, leading to the leaching of compounds, consequently increasing the SB.

On the contrary, the pasting viscosity of the WR subjected to the RF treatment significantly increased compared to the control. As shown in Table 2, the PV, TV, SB, BD, and FV of the RF-treated WR were higher than untreated samples. The elevated PV may be associated with the destruction of starch granules and the damage of the microstructure (crystalline and helical structure), which favored the granules swelling and raised the stretching of starch molecules. Additionally, the increased PV may be due to the interactions between water and stretched molecules, which formed intra- and inter-hydrogen bonds and networked chains [28]. In addition, the BV represents the heat and shear resistance of starch granules at high temperatures. The greater BV implied that the RF treatment reduced the thermal stability of starch paste. The higher SB of the RF-treated WR may be due to the degradation of amylopectin, leading to the rearrangement of starch chains. The different changes in the RF-treated rice grains could be related to their varied compositions and structures.

### 3.6. Rheological Characterization

**Figure 5:**
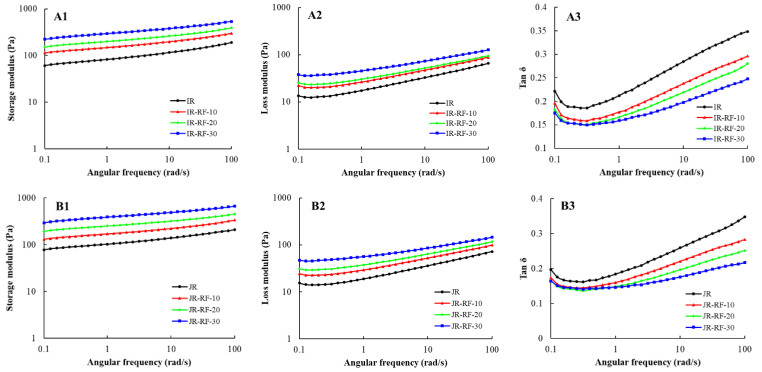

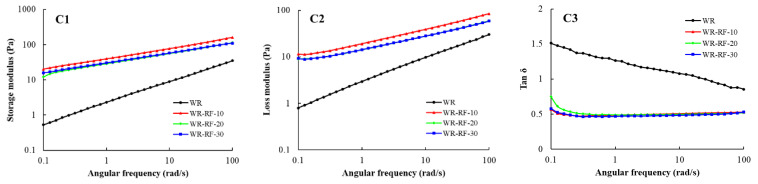
The dynamic viscoelastic properties of untreated and RF-treated rice. (**A1**–**A3**): storage modulus (G′); (**B1**–**B3**): loss modulus (G″); (**C1**–**C3**): tan δ; IR: indica rice; JR: japonica rice; WR: waxy rice.

Understanding the rheological properties of rice gels is instructive for the production of gluten-free products. The rheological behavior of rice gels formed by the RF-treated samples is presented in Figure 5. The G′ and G″ of all samples displayed a raised trend with the increasing angular frequency. The G′ values were higher than the G″ values for all samples, except for the native WR, meaning that these gels exhibited weak gel behaviors. Conversely, the native WR displayed a dominant viscous property (G′ < G″). Moreover, both the G′ and G″ of rice grains after the RF treatment significantly increased compared with the control, meaning that the RF treatment enhanced the strength of the cross-linked gel network. As confirmed by RVA, the setback values of the RF-treated samples increased. It showed that the RF treatment accelerated the retrogradation of rice gels and improved their gelling ability [12]. Similarly, Solaesa et al. [32] found that the microwave treatment markedly enhanced the G′ and G″ of rice flour. They confirmed that gels created from MV-treated flour were more stable. In addition, the tan δ of all RF-treated gels was obviously reduced compared to those of untreated gels, implying that the tightness and stability of the internal structure of rice grains were destroyed after the RF treatment [22]. With the increase in angular frequency, the loss tangent (tan δ) of IR and JR before and after the RF treatment firstly decreased (0.1–0.5 rad/s) and then increased. The relative tan δ values were less than one, implying that the IR and JR gels displayed a solid-like behavior. On the other hand, the tan δ of the untreated WR exhibited a downward trend with the increasing angular frequency, while the tan δ of the RF-treated WR first decreased and then slightly increased. The gels created from the untreated WR exhibited a higher tan δ value (tan δ > 1) at a lower frequency (0.1–20 rad/s), suggesting that the gels showed more viscous characteristics, whereas the gels presented a more solid-like behavior (tan δ < 1) at a higher frequency (20–100 rad/s). Moreover, the RF-treated WR gels differed from the control, which showed a more solid-like behavior (tan δ < 1). These differences among the three rice varieties could be associated with changes in other components of rice, such as protein, lipids, and phenolic acids [17].

### 3.7. In Vitro Digestibility of Rice

The digestibility of rice grains subjected to the RF treatment was assessed with the starch fractions. Thereby, the results of RDS, SDS, and RS are summarized in Table 2. The digestibility of rice grains after the RF treatment was altered to various degrees, especially for the RDS and RS contents. Compared with native IR and JR, the RF treatment led to a decrease in the RDS content and an increase in the RS fraction. The increased RS indicated that the RF treatment reinforced the interaction between the starch and lipid, thus, inhibiting the physical accessibility to enzymes. Cheng et al. [36] suggested that the formation of amylose–lipid complexes after the heat moisture treatment enhanced the RS content and reduced the swelling and digestibility of starch. Moreover, the protein could have been denatured during the RF treatment and adhered to the surface of starch granules [37], which restrained the susceptibility of starch to enzymes. However, the RF-treated WR had lower SDS and RS contents than those in the native sample, and this fraction could be transformed into RDS. This phenomenon could be due to the complete gelatinization of starch molecules, as confirmed by the SEM and DSC results. The differences in the digestibility of the three rice varieties depended on many factors, such as the amylose/amylopectin content, chemical components (proteins and lipids), starch granular structure, and experiment conditions [30,38]. It has been reported that the amylopectin chain length influences the formation of crystalline starch polymorphs, thereby affecting the digestibility of starch [39].

### 3.8. Mechanism of RF Treatment for Rice

In this study, we discovered that the RF treatment had the potential to improve the structural, physicochemical, and digestive properties of rice grains. During the RF treatment, the friction and collision among polar molecules rapidly produced more heat in rice grains, thereby facilitating the interactions between starch chains or starch and other components. The formation of starch–lipid complexes increased the RS content and reduced the starch digestibility. The RF treatment promoted the formation of protein gels network, which restrained the contact between starch and enzymes. Moreover, the RF treatment improved the stability of rice grains as reflected by the enhancement of the gelatinization temperature (T_o_, T_p_ and T_c_). Moreover, the RF treatment destroyed the crystalline and short-range ordered structures of starch granules, and changed the microstructure of endosperm cells. The chemical and structural changes in turn altered the physicochemical properties of rice grains.

## 4. Conclusions

The microstructures, physicochemical properties, and digestibility of rice grains were altered after the RF treatment. In terms of the RF-treated IR and JR, the formation of amylose–lipid complexes contributed to the increase in the RS content, thereby reducing the digestibility. Moreover, the enhanced T_o_, T_p_, and T_c_ values of the RF-treated IR and JR suggested that new aggregation structures formed during the RF treatment, which was also conducive to reducing the digestibility. In addition, with the increase in treatment time, the RF treatment resulted in the gelatinization of starch molecules, the destruction of the crystalline structure, and the destruction of short-range ordered structures to different degrees. Furthermore, the RF treatment improved the physicochemical properties (pasting and rheology) of rice gains. These results confirmed that the RF treatment exhibits the potential for modifying the digestibility of rice. However, the effects of the RF treatment for the cooking time and quality of rice compared to the conventional method remain unclear, and should be studied in future work.

## Figures and Tables

**Table 1:** The X-ray diffraction, FTIR, and thermal characteristics of native and RF-treated rice grains.

Samples	RC (%)	R_1022/995_	R_1048/1022_	T_o_	T_p_	T_c_	ΔH
IR	25.7 ± 0.8 ^bc^	0.98 ± 0.009 ^d^	0.71 ± 0.006 ^abc^	65.3 ± 0.07 ^c^	71.8 ± 0.1 ^d^	78.5 ± 0.5 ^c^	3.2 ± 0.2 ^b^
IR-RF-10	21.6 ± 0.6 ^cd^	1.28 ± 0.009 ^abc^	0.67 ± 0.001 ^bc^	72.1 ± 0.8 ^ab^	77.1 ± 0.5 ^a^	81.5 ± 1.8 ^abc^	0.8 ± 0.1 ^cd^
IR-RF-20	22.1 ± 2.5 ^cd^	1.35 ± 0.03 ^a^	0.62 ± 0.01 ^c^	71.9 ± 0.05 ^ab^	77.4 ± 0.2 ^a^	83.1 ± 0.7 ^ab^	1.3 ± 0.2 ^c^
IR-RF-30	20.5 ± 2.1 ^d^	1.35 ± 0.03 ^ab^	0.69 ± 0.003 ^bc^	72.6 ± 0.3 ^a^	78.1 ± 0.1 ^a^	83.9 ± 0.3 ^a^	1.3 ± 0.1 ^c^
JR	28.3 ± 1.3 ^b^	1.02 ± 0.004 ^d^	0.71 ± 0.002 ^abc^	59.6 ± 0.07 ^d^	66.4 ± 0.1 ^e^	73.1 ± 0.3 ^d^	4.1 ± 0.5 ^b^
JR-RF-10	20.9 ± 0.8 ^cd^	1.25 ± 0.02 ^bc^	0.69 ± 0.02 ^bc^	68.1 ± 3.2 ^bc^	74.4 ± 0.7 ^bc^	80.9 ± 1.5 ^abc^	1.1 ± 0.2 ^c^
JR-RF-20	21.3 ± 1.6 ^cd^	1.27 ± 0.02 ^abc^	0.69 ± 0.006 ^bc^	68.7 ± 1.1 ^abc^	73.7 ± 0.2 ^c^	79.1 ± 0.5 ^c^	0.8 ± 0.2 ^cd^
JR-RF-30	19.5 ± 0.5 ^d^	1.29 ± 0.05 ^abc^	0.65 ± 0.04 ^bc^	69.9 ± 0.9 ^ab^	75.3 ± 0.3 ^b^	79.8 ± 1.1 ^bc^	0.6 ± 0.3 ^cd^
WR	37.0 ± 0.7 ^a^	0.98 ± 0.01 ^d^	0.74 ± 0.02 ^ab^	58.5 ± 0.5 ^d^	65.7 ± 0.6 ^e^	73.2 ± 1.1 ^d^	5.5 ± 0.4 ^a^
WR-RF-10	7.38 ± 0.7 ^e^	1.21 ± 0.07 ^c^	0.79 ± 0.04 ^a^	-	-	-	-
WR-RF-20	7.10 ± 0.1 ^e^	1.22 ± 0.004 ^c^	0.74 ± 0.01 ^ab^	-	-	-	-
WR-RF-30	4.35 ± 0.3 ^e^	1.24 ± 0.05 ^c^	0.73 ± 0.02 ^ab^	-	-	-	-

Different letters in the same column indicate a significant difference (*p* < 0.05). IR: indica rice; JR: japonica rice, WR: waxy rice; RF: radio frequency.

**Table 2:** The pasting and in vitro digestibility properties of rice grains subjected to RF treatment.

Samples	PV (Pa·s)	BD (Pa·s)	T (Pa·s)	FV (Pa·s)	SB (Pa·s)	RDS (%)	SDS (%)	RS (%)
IR	3238 ± 4.9 ^a^	1939 ± 9.9 ^a^	1299 ± 4.9 ^bc^	2371 ± 3.54 ^e^	1072 ± 1.4 ^d^	63.8 ± 0.8 ^abc^	3.2 ± 1.2 ^a^	32.8 ± 0.4 ^ab^
IR-RF-10	2600 ± 30.4 ^b^	979 ± 7.8 ^c^	1621 ± 22.6 ^a^	2897 ± 21.9 ^b^	1276 ± 0.7 ^b^	59.8 ± 1.0 ^bc^	5.1 ± 0.2 ^a^	34.9 ± 0.8 ^ab^
IR-RF-20	2192 ± 46.7 ^de^	564 ± 19.8 ^de^	1628 ± 26.9 ^a^	3071 ± 24.0 ^a^	1443 ± 2.8 ^a^	59.5 ± 3.5 ^bc^	4.8 ± 1.5 ^a^	35.5 ± 0.1 ^ab^
IR-RF-30	1379 ± 1.4 ^g^	112 ± 12.0 ^f^	1266 ± 10.6 ^c^	2435 ± 22.6 ^de^	1168 ± 33.2 ^c^	60.7 ± 1.4 ^abc^	4.5 ± 1.0 ^a^	34.6 ± 0.4 ^ab^
JR	2354 ± 14.8 ^cd^	1319 ± 27.6 ^b^	1035 ± 12.7 ^e^	2100 ± 14.8 ^g^	1065 ± 2.1 ^d^	64.3 ± 0.6 ^abc^	1.9 ± 1.0 ^a^	33.7 ± 1.6 ^ab^
JR-RF-10	2035 ± 46.0 ^e^	735 ± 27.6 ^d^	1300 ± 18.4 ^bc^	2485 ± 25.5 ^d^	1185 ± 7.1 ^c^	60.1 ± 2.3 ^abc^	1.3 ± 0.6 ^a^	38.4 ± 1.6 ^a^
JR-RF-20	1809 ± 13.4 ^f^	468 ± 4.2 ^e^	1341 ± 17.7 ^b^	2588 ± 24.0 ^c^	1246 ± 6.4 ^b^	57.6 ± 2.9 ^c^	7.6 ± 2.3 ^a^	34.6 ± 0.4 ^ab^
JR-RF-30	1316 ± 24.0 ^g^	164 ± 6.3 ^f^	1151 ± 17.7 ^d^	2198 ± 36.1 ^f^	1047 ± 18.4 ^d^	60.6 ± 0.4 ^abc^	1.3 ± 0.6 ^a^	38.0 ± 1.0 ^a^
WR	1335 ± 28.7 ^g^	1000 ± 44.0 ^c^	335 ± 14.8 ^f^	467 ± 20.5 ^k^	132 ± 5.7 ^g^	57.8 ± 1.4 ^c^	4.4 ± 1.2 ^a^	37.7 ± 0.2 ^a^
WR-RF-10	2359 ± 20.5 ^cd^	1243 ± 35.4 ^b^	1116 ± 14.8 ^d^	1612 ± 14.1 ^j^	495 ± 0.7 ^f^	65.0 ± 0.8 ^ab^	3.2 ± 2.0 ^a^	31.6 ± 1.2 ^b^
WR-RF-20	2518 ± 7.1 ^bc^	1347 ± 2.8 ^b^	1171 ± 4.2 ^d^	1714 ± 17.0 ^i^	543 ± 12.7 ^ef^	67.1 ± 1.2 ^a^	2.3 ± 0.8 ^a^	30.5 ± 2.0 ^b^
WR-RF-30	2618 ± 10.6 ^b^	1326 ± 14.1 ^b^	1292 ± 3.5 ^bc^	1879 ± 4.24 ^h^	586 ± 0.7 ^e^	62.1 ± 2.0 ^abc^	2.6 ± 1.2 ^a^	35.2 ± 3.3 ^ab^

Different letters in the same column indicate a significant difference (*p* < 0.05). IR: indica rice; JR: japonica rice; WR: waxy rice; RF: radio frequency.

## Data Availability

Research data are not shared.
